# A novel inflammation-nutrition risk score (INRS) and its related nomogram model to predict radiological bronchiectasis in patients after tuberculosis infection in Wuhan, China

**DOI:** 10.1080/07853890.2026.2625545

**Published:** 2026-02-17

**Authors:** Qi Yu, Jisong Yan, Jianping Song, Fan Yu, Nanchuan Jiang, Yaya Zhou, Xinliang He, Fengyun Gong, Xiaorong Wang

**Affiliations:** ^a^ Department of Gastroenterology, Union Hospital, Tongji Medical College, Huazhong University of Science and Technology, Wuhan, China; ^b^ Department of Respiratory and Critical Care Medicine, Union Hospital, Tongji Medical College, Huazhong University of Science and Technology, Wuhan, China; ^c^ Department of lnfectious Diseases, Wuhan Jinyintan Hospital, Tongji Medical College, Huazhong University of Science and Technology, Wuhan, China; ^d^ National Center for Respiratory Diseases, State Key Laboratory of Respiratory Health and Multimorbidity, National Clinical Research Center for Respiratory Diseases, Institute of Respiratory Medicine, Chinese Academy of Medical Sciences, Department of Pulmonary and Critical Care Medicine, Center of Respiratory Medicine, China-Japan Friendship Hospital, Beijing, China; ^e^ Chinese Academy of Medical Sciences & Peking Union Medical College, Beijing, China; ^f^ Department of Radiology, Union Hospital, Tongji Medical College, Huazhong University of Science and Technology, Wuhan, China

**Keywords:** Radiological bronchiectasis, tuberculosis infection, inflammation-nutrition risk score, nomogram model

## Abstract

**Background:**

Tuberculosis infection (TBI) is a significant cause of bronchiectasis (BE). Identifying risk factors for radiological BE (RBE) could enhance the early detection of high-risk individuals following TB infection. This study aimed to develop and validate a novel Inflammation-Nutrition Risk Score (INRS) and a corresponding nomogram model to predict the risk of RBE after TBI.

**Patients and methods:**

We enrolled 2,210 post-TBI patients from two medical centres. Data from 1,825 patients at Wuhan Jinyintan Hospital were used to develop the INRS and the RBE nomogram. An independent cohort of 385 patients from Wuhan Union Hospital served as an external validation set.

**Results:**

The INRS was derived from four parameters: PNI, HALP score, Lg(SII) and CAR. Multivariate analysis identified the following independent risk factors for RBE: age ≥60 years (OR = 1.19, *p* = 0.030), current smoking (OR = 1.71, *p* = 0.009), COPD (OR = 3.13, *p* < 0.001), RDW-CV ≥12.8% (OR = 1.09, *p* = 0.005), ALB <35.5 g/L (OR = 1.04, *p* = 0.003) and INRS ≥1.86 (OR = 5.04, *p* < 0.001). The RBE nomogram model demonstrated strong discriminatory power, accuracy and clinical utility across the development, internal validation and external validation cohorts.

**Conclusion:**

In post-TBI patients, the INRS represents a novel predictive biomarker for RBE. The INRS-based nomogram is a clinically applicable and efficient tool for risk stratification and guiding follow-up management to prevent RBE progression.

## Introduction

Bronchiectasis (BE) is characterized by irreversible pathological dilation of the bronchi, which may present with clinical symptoms such as chronic cough and sputum production, or remain asymptomatic [1,2]. Although large-scale global epidemiological data are limited, studies from various countries indicate a rising incidence and prevalence of BE [3–8], accompanied by a growing socioeconomic [9] and public health burden, including increased mortality and hospital admissions [3,5]. The aetiology of BE is diverse, encompassing cystic fibrosis, humoral immunodeficiency, and post-infectious sequelae, among others [9–14]. Among known causes, post-infection – particularly following tuberculosis (TB) – is the most common, aside from idiopathic BE [15,16]. A real-world observational study highlighted that post-TB bronchiectasis occurs most frequently in Asian countries, especially those with a high TB burden [17].

Studies on post-TB lung disease report that approximately 40% of patients who complete TB treatment develop moderate-to-severe BE [18, 19]. However, BE may sometimes be solely a radiographic finding, termed radiological BE (RBE), which is often associated with minimal or no symptoms or exacerbations [2,20]. A study from India found that about 40% of TB survivors develop RBE [16]. Currently, the long-term clinical outcomes of RBE following TB infection remain unclear. Our previous research demonstrated that patients with rifampicin- or multidrug-resistant TB and concomitant RBE had significantly lower survival over a 2-year follow-up compared to those without RBE [21]. Furthermore, in an on-going study, we observed that 20.27% of 296 initially asymptomatic RBE patients progressed to clinical BE (CBE) within 4 years, suggesting that RBE may represent an early, pre-symptomatic stage of CBE, with progression driven by various risk factors. Therefore, early prevention of RBE – starting from the stage when no radiographic changes are evident – is crucial to improving long-term prognosis and quality of life in post-TB individuals. Nevertheless, data on the prevalence, risk factors and management of RBE following TB infection remain scarce.

Given this gap, this study aimed to investigate the prevalence of RBE in post-TB patients in Wuhan, China. Furthermore, we sought to identify risk factors for RBE and to develop a novel Inflammation‑Nutrition Risk Score (INRS) and a corresponding nomogram model for early prediction of RBE. This model could facilitate the identification of high-risk individuals and support the formulation of personalized management strategies, thereby contributing to precision medicine in post-TB care.

## Material and methods

### Study participants and design

This multicentre, retrospective study enrolled patients with a history of tuberculosis infection (TBI) from two centres: Wuhan Jinyintan Hospital (Wuhan Infectious Disease Hospital) from January 2015 to January 2022, and Wuhan Union Hospital from January 2022 to June 2023. All patients were affiliated with Tongji Medical College, Huazhong University of Science and Technology.

Based on the international consensus criteria for radiological bronchiectasis (RBE) [2], patients were categorized into an RBE group and a non-RBE (NRBE) group. From the Wuhan Jinyintan Hospital cohort, patients were randomly allocated to a development set (70%) for constructing the Inflammation-Nutrition Risk Score (INRS) and nomogram model, and an internal validation set (30%) for initial validation. Patients from Wuhan Union Hospital constituted an independent external validation set.

### Inclusion and exclusion criteria

Inclusion criteria were: (1) a past history of confirmed pulmonary tuberculosis (positive culture and/or Xpert MTB/RIF assay) who had completed anti-tuberculosis therapy and were in a stable clinical state at the time of assessment. (2) For the RBE group, meeting the diagnostic imaging criteria for RBE as per the international consensus [2]: (a) few or no symptoms associated with clinical bronchiectasis; (b) internal airway-to-artery diameter ratio ≥1.0; (c) lack of bronchial tapering and (d) Airway visibility within 1 cm of costal pleural surface or touching mediastinal pleura.

Exclusion criteria were: (1) age <18 years; (2) unavailable key information and (3) for the RBE group: (a) a prior diagnosis of RBE or CBE before the index TBI episode, based on medical history and radiological records; (b) bronchiectasis due to cystic fibrosis, or traction bronchiectasis secondary to interstitial lung disease or other respiratory disorders.

This study was conducted in line with the Declaration of Helsinki. The institutional ethics committees of Wuhan Jinyintan (KY-2022-06.01) and Wuhan Union Hospital (No.2022-0455-01) reviewed and approved this study protocol. Informed consent was obtained from all patients or next kin for this study.

### Variable collection and quality control

All data were extracted from the unified electronic medical record (EMR) systems of the two hospitals to minimize manual recording bias. Collected variables included demographics (age, gender, weight, education), treatment history, smoking and alcohol use, comorbidities (diabetes, hypertension, hepatitis B virus), underlying pulmonary diseases (chronic pulmonary heart disease, COPD), radiological features (the presence of RBE) and laboratory indices.

We computed multiple inflammatory and nutritional indices, including the Systemic Inflammation Index (SII), Systemic Inflammatory Response Index (SIRI), neutrophil-to-lymphocyte ratio (NLR), derived NLR (dNLR), monocyte-to-lymphocyte ratio (MLR), platelet-to-lymphocyte ratio (PLR), neutrophil-to-high-density-lipoprotein ratio (NHR), monocyte-to-high-density-lipoprotein ratio (MHR), platelet-to-high-density-lipoprotein ratio (PHR), monocyte-to-red-blood-cell ratio (MRR), C-reactive protein-to-albumin ratio (CAR), C-reactive protein-to-pre-albumin ratio (CPR), C-reactive protein-to-lymphocyte ratio (CLR), haemoglobin-albumin-lymphocyte-platelet (HALP) score, prognostic nutritional index (PNI) and controlling nutritional status (CONUT) score. Detailed formulas are provided in Supplementary Table 1.

Multi-stage quality control measures in this study including: (1) Double-Independent Data Extraction: Two trained assistants independently extracted data using standardized forms. Inter-rater agreement was high (Cohen’s kappa = 0.92, 95% CI: 0.89–0.95 for categorical variables; intraclass correlation coefficient = 0.96, 95% CI: 0.94–0.98 for continuous variables). (2) Abnormal Value Verification: Outliers (beyond 3 × IQR) flagged by R software (v4.2.1) were cross-referenced with original EMRs and reviewed by a senior clinician (X.H., ≥10 years of experience). (3) Final Data Audit: An independent data monitoring committee reviewed the final dataset prior to analysis. (4) Standardized Timing: Data collection was temporally aligned: (a) Baseline data: within 24 h of admission; (b) laboratory indices: within 48 h of admission (pre-treatment); (c) chest CT for RBE diagnosis: within 1 week of laboratory draw.

### Statistical analysis

Categorical data are presented as *n* (%) and compared using the Chi-square or Fisher’s exact test. Continuous data are presented as mean ± standard deviation (SD) and compared using Student’s t-test.

**(1) Missing value handling:** Prior to analysis, the missing rate of each variable was evaluated. Variables with a missing rate <20% were imputed using multiple imputation techniques to mitigate potential bias caused by missing data. Specifically, 5 imputed data sets (*m* = 5) were generated based on the predictive mean matching method, with imputation models incorporating all covariates and the outcome variable (RBE) to ensure robustness. The imputed data sets were combined using Rubin’s rules to generate final parameter estimates and confidence intervals. Variables with a missing rate ≥20% were excluded from subsequent analyses due to insufficient information.

**(2) Collinearity diagnosis:** Given the potential mathematical correlation and redundancy among predictors (including base laboratory indicators and composite indices), formal collinearity diagnosis was performed prior to nomogram construction. ①A Pearson correlation matrix of all candidate variables (including inflammation/nutrition indicators, INRS and other baseline covariates) was generated, and pairwise correlation coefficients (r) were reported; variables with |r| > 0.7 were flagged as highly correlated. ② Variance Inflation Factor (VIF) was calculated using the car package (v3.1-1) of R software: A VIF value >10 indicated severe multicollinearity, >5 indicated moderate multicollinearity and <3 indicated no significant multicollinearity. For variables with severe multicollinearity, only the variable with the strongest clinical relevance or highest predictive power (based on ULSA results) was retained to avoid redundant information.

**(3) Construction, evaluation and validation of INRS:** In the development set, INRS was constructed by Univariate and Multivariate Logistic regression analysis (ULSA and MLSA) based on inflammation and nutrition indicators with statistically significant differences. Subsequently, the receiver operating characteristics (ROC) analysis and multivariate logistic regression analysis were used to evaluate the prediction performance and stability of INRS for RBE. In addition, the internal and external sets were used to validate the INRS.

**(4) Construction, evaluation and validation of the RBE nomogram model:** After collinearity diagnosis, INRS and remaining variables with *p* < 0.05 in ULSA were included in Least Absolute Shrinkage and Selection Operator (LASSO) regression (10-fold cross-validation *via* glmnet package v4.1-6) for further variable selection in the development set, which further mitigated multicollinearity and overfitting risks. Optimal predictors were then identified *via* MLSA to construct the RBE nomogram model. Model performance was comprehensively evaluated using: ① ROC analysis (DeLong test for AUC comparison between training and validation sets); ② calibration curve analysis (CCA) and calculation of external calibration slope/intercept and ③ decision curve analysis (DCA) to assess clinical utility by quantifying net benefit across different threshold probabilities. If miscalibration was detected in external validation, recalibration was performed, and the performance of the recalibrated model was re-evaluated. Internal validation set and external validation set were performed in the independent external set to confirm the model’s generalizability.

All analyses were performed using SPSS 26.0 (IBM Corp., Armonk, NY, USA) and R software (R Foundation for Statistical Computing, Vienna, Austria). The mice package in R was used for multiple imputation, the pROC package for ROC analysis and DeLong test, and the rms package for nomogram construction and calibration analysis. A two-sided *p*-value <0.05 was considered statistically significant. A previous version of this manuscript was published as a preprint [22].

## Results

### Study population and characteristics

**Figure 1.**
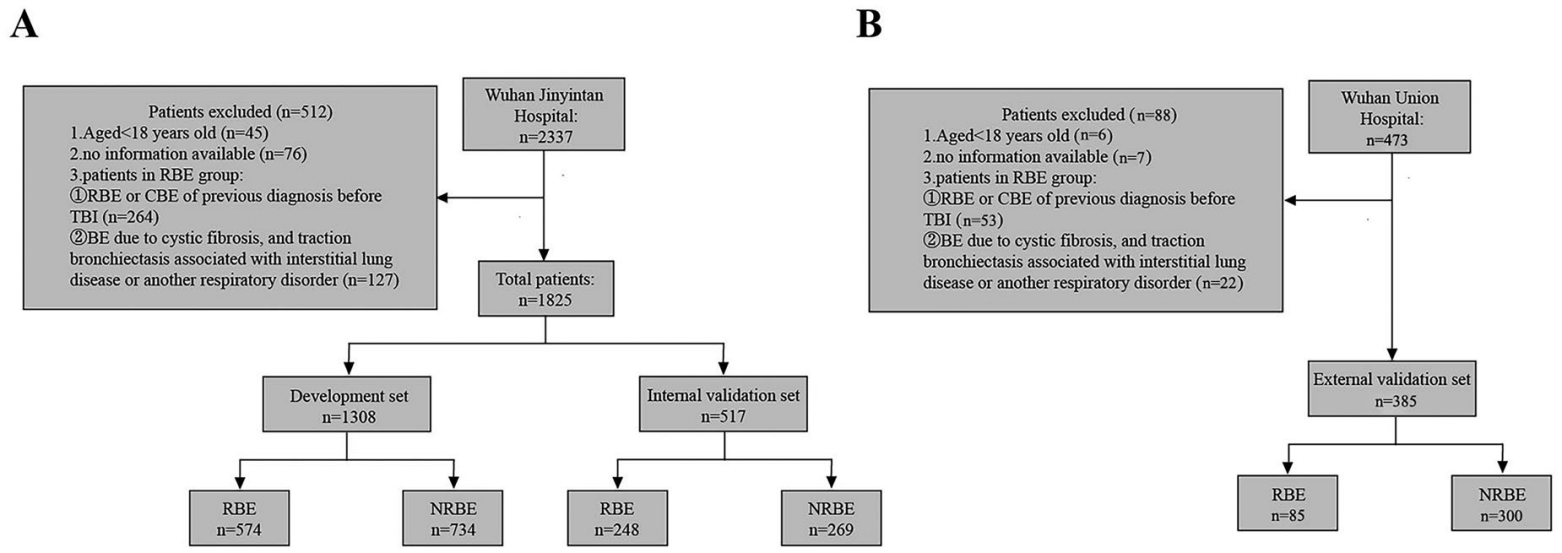
The selection flowchart of patients after TBI: (A) Wuhan Jinyintan Hospital; and (B) Wuhan Union Hospital.

A total of 1,825 post-TBI patients from Wuhan Jinyintan Hospital were included. Of these, 1,308 patients (70%) were randomly allocated to the development set, and 517 (30%) constituted the internal validation set. Additionally, 385 patients from Wuhan Union Hospital formed the independent external validation set. The patient selection flow is detailed in Figure 1, and the baseline characteristics of the three cohorts are summarized in Table 1.

**Table 1.** Baseline characteristics among development set, internal validation set and external validation set.

Characteristics	Development set (*n* = 1308)	Internal validation set (*n* = 517)	External validation set (*n* = 385)	*p*
**Age**	47.34 ± 17.38	48.31 ± 17.58	52.26 ± 12.47	<0.001
**Gender**				0.258
Male	940 (71.87%)	354 (68.47%)	265(68.83)	
Female	368 (28.13%)	163 (31.53%)	120(31.17)	
**Weight**	55.51 ± 9.84	55.02 ± 10.20	56.95 ± 8.53	0.002
**Smoking**				<0.001
Somker	367 (28.06%)	139 (26.89%)	83(21.56)	
Ex-smoker	242 (18.5%)	87 (16.83%)	64(16.62)	
Never	699 (53.44%)	291 (56.29%)	238(61.82)	
**Drink**				0.006
Drinker	164 (12.54%)	61 (11.8%)	30(7.79)	
Ex-drinker	107 (8.18%)	52 (10.06%)	24(6.23)	
Never	1037 (79.28%)	404 (78.14%)	330(85.71)	
**HTN**	139 (10.63%)	57 (11.03%)	59(15.32)	0.037
**DM**	244 (18.65%)	108 (20.89%)	60(15.58)	0.129
**CPDH**	122 (9.33%)	36 (6.96%)	27(7.01)	0.148
**COPD**	337 (25.76%)	124 (23.98%)	79(20.52)	0.105
**HBV**	185 (14.14%)	62 (11.99%)	37(9.61)	0.052
**RBE**	574 (43.88%)	248 (47.97%)	85(22.08)	<0.001

**Abbreviation:** HTM, Hypertension; DM = Diabetes Melitus; CPDH, Chronic pulmonary heart disease; COPD, Chronic obstructive pulmonary disease; HBV, Chronic hepatitis B; RBE, Radiological bronchiectasis.

Within the development set, significant differences (*p* < 0.05) were observed between the RBE and NRBE groups across a range of demographic, clinical and laboratory variables, including age, smoking history, CPHD, COPD and multiple inflammatory–nutritional indices (e.g. PNI, HALP, SII, CAR). Variables with no significant difference (*p* > 0.05) are listed in Supplementary Table 2.

### Collinearity diagnosis

Formal collinearity diagnosis was performed for all candidate variables (including baseline laboratory indicators, inflammatory-nutritional indices and composite indices) prior to model construction. Pearson correlation analysis revealed that the pairwise correlation coefficient (r) of some variables > 0.7, indicating severe bivariate collinearity (Supplementary Figure 1). For variables with severe collinearity (*r* ≥ 0.7), a prioritization strategy was adopted to eliminate redundancy: the variable with the highest contribution to the model was retained, while the other correlated variables were excluded. The ‘highest contribution’ was defined based on three criteria: ① clinical relevance (priority to indicators with well-established associations with RBE in existing literature); ② statistical significance (lowest *p* value in univariate logistic regression) and ③ effect size (the largest absolute value of the odds ratio in univariate analysis). After this collinearity handling, the remaining variables had pairwise correlation coefficients (*r*) < 0.7 and VIF values < 5 (Supplementary Tables 3 and 4), confirming the absence of significant multicollinearity. These results ensured that multicollinearity would not substantially impact the subsequent regression analyses, and the refined set of candidate variables was retained for further selection.

**Table 3.** Logistic regression analysis for the factors of RBE selected by LASSO regression training set.

	Univariate		Multivariate	
	OR (95% CI)	*p* value	OR (95% CI)	*p* value
Age				
< 60 years old	Ref.	–	Ref.	–
≥ 60 years old	1.76 (1.38–2.25)	<0.001	1.19 (1.05-1.78)	0.030
Smoking status				
No	Ref.	–	Ref.	–
Ex-smoker	1.11 (0.86–1.44)	0.419	0.94 (0.68-1.30)	0.712
Smoker	1.79 (1.34–2.41)	<0.001	1.71 (1.14-2.56)	0.009
COPD				
No	Ref.	–	Ref.	–
Yes	3.46 (2.66–4.49)	<0.001	3.13 (2.30–4.26)	<0.001
RDW	1.18 (1.10–1.26)	<0.001	1.09 (1.03–1.16)	0.005
ALB	0.95 (0.93–0.97)	<0.001	1.04 (1.01–1.06)	0.003
BUN	1.01 (0.98–1.05)	0.440		
Scr	0.99 (0.98–0.99)	0.047	1.00 (0.99–1.00)	0.065
eGFR	1.00 (0.99–1.01)	0.343		
Na	0.98 (0.96–1.01)	0.329		
APTT	1.00 (0.98–1.01)	0.703		
TC	0.98 (0.87–1.12)	0.855		
PCT	5.28 (1.17–23.95)	0.031	0.44 (0.08–2.50)	0.356
SAA	1.00 (0.99–1.01)	0.092		
Risk score				
Low-risk group	Ref.	–	Ref.	–
High-risk group	4.74 (3.75–6.00)	<0.001	5.04 (3.79–6.70)	<0.001

**Abbreviation**: OR, odds ratio, 95%CI, 95% confidence interval.

### Development, evaluation, and validation of the INRS

As shown in Table 2, univariate and multivariate logistic regression identified four key indicators for constructing the Inflammation-Nutrition Risk Score (INRS): PNI (OR = 0.95, 95% CI: 0.91–0.99), HALP score (OR = 0.97, 95% CI: 0.96–0.98), Lg(SII) (OR = 3.99, 95% CI: 1.07–14.85) and CAR (OR = 1.15, 95% CI: 1.06–1.25). The resulting formula was: INRS = −0.051 × PNI − 0.031 × HALP + 1.385 × Lg(SII) + 0.142 × CAR, with an optimal ROC-derived cut-off value of 1.86.

**Table 2.** Logistic regression analysis of inflammatory indicators for RBE in the development set.

	Univariate			Multivariate		
	β	OR (95%CI)	*p*	β	OR (95%CI)	*p*
PNI	−0.035	0.97 (0.95–0.98)	<0.001	−0.051	0.95 (0.91–0.99)	0.030
HALP	−0.017	0.98 (0.97–0.99)	<0.001	−0.031	0.97 (0.96–0.98)	0.012
COUNT	0.080	1.08 (1.04–1.13)	<0.001			
SII	0.000	1.00 (1.00–1.00)	0.235			
Lg (SII)	0.634	1.89 (1.43–2.48)	<0.001	1.385	3.99 (1.07–14.85)	0.039
SIRI	0.008	1.01 (0.99–1.02)	0.273			
NLR	0.004	1.00 (1.00–1.01)	0.421			
Lg (NLR)	0.607	1.84 (1.32–2.55)	<0.001			
MLR	0.201	1.22 (0.98–1.52)	0.072			
PLR	0.000	1.00 (1.00–1.00)	0.217			
Lg (PLR)	0.729	2.07 (1.39–3.09)	<0.001			
dNLR	0.020	1.02 (0.98–1.05)	0.205			
NHR	0.039	1.04 (1.02–1.06)	0.001			
MHR	0.460	1.58 (1.20–2.09)	0.001			
pP	0.001	1.00 (1.00–1.00)	<0.001			
Lg (pP)	0.721	2.06 (1.40–3.02)	<0.001			
MRR	3.113	22.49 (4.41–114.70)	<0.001			
CAR	0.195	1.22 (1.13–1.31)	<0.001	0.142	1.15 (1.06–1.25)	<0.001
CPR	0.069	1.07 (1.01–1.15)	0.048			
CLR	0.001	1.00 (1.00–1.00)	0.174			
Lg (CLR)	0.405	1.50 (1.29–1.75)	<0.001			

**Abbreviation:** INRS = -0.051*PNI -0.031*HALP + 1.385*Lg(SII)+0.142*CAR.

ROC cut-off: 1.86.

**Figure 2.**
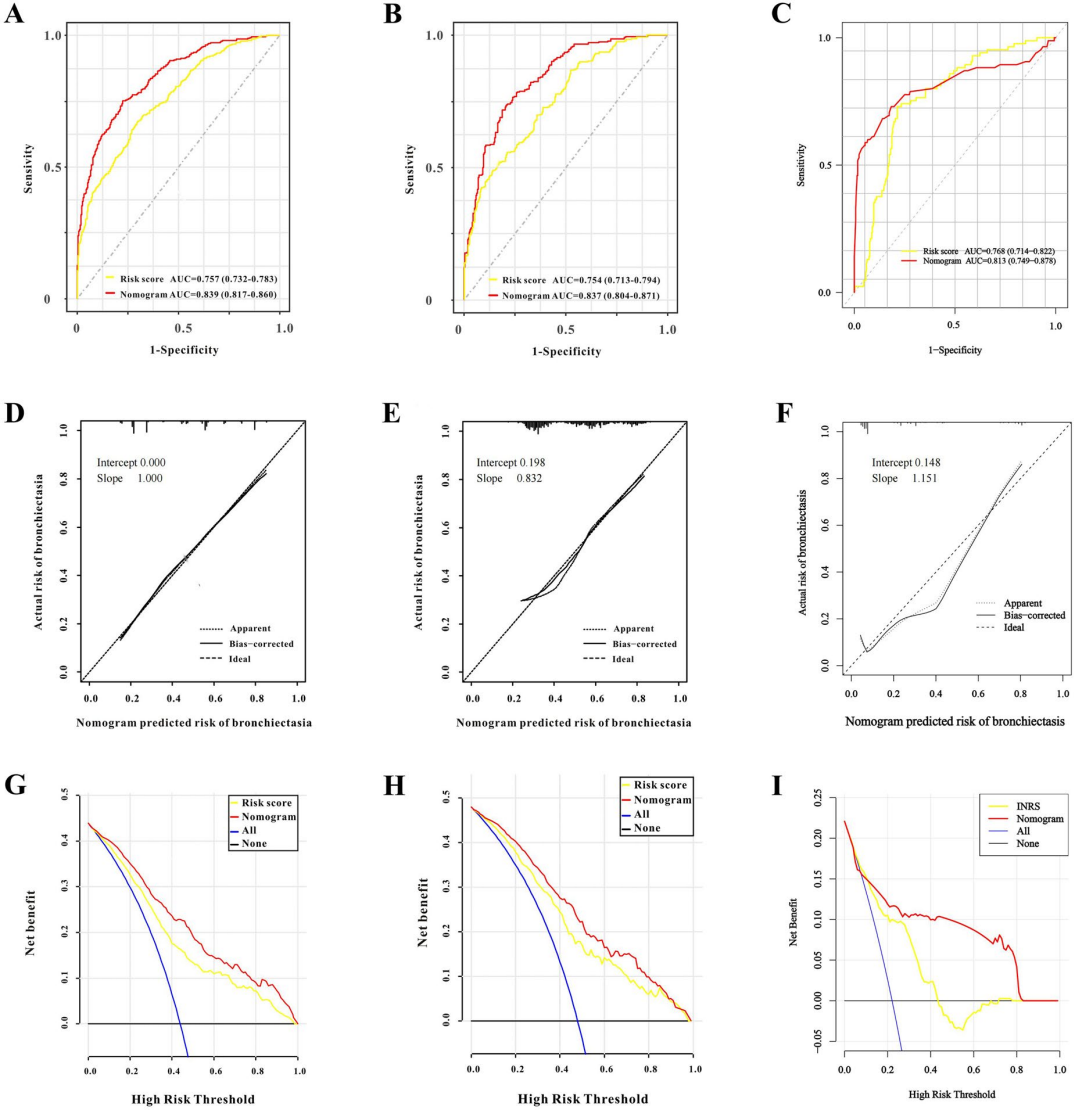
The evaluation and validation for the INRS and RBE nomogram model. (A-C) the ROC curve for the INRS and RBE nomogram model; (D-F) the calibrate curve for the RBE nomogram model; (G-I) the decision curve for the RBE nomogram model.

**Figure 3.**
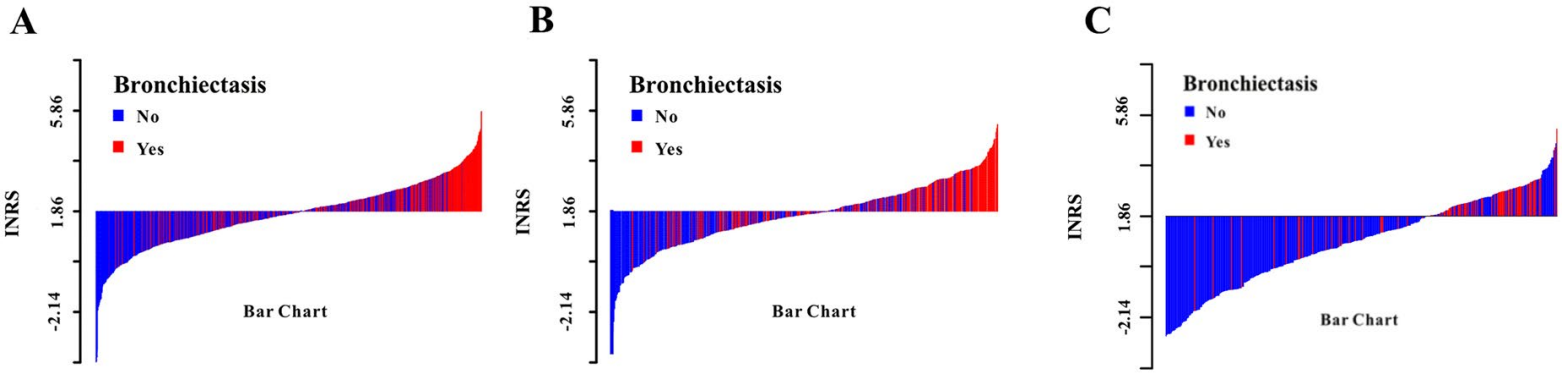
The RBE distribution in patients after TBI between high INRS and low INRS. (A) Development set; (B) Internal validation set; (C) External validation set.

The INRS demonstrated consistent predictive performance across all cohorts, with AUCs of 0.757 (development set; Figure 2A), 0.754 (internal validation; Figure 2B) and 0.761 (external validation; Figure 2C). After adjusting for potential confounders including age, gender, weight, smoking, alcohol use, comorbidities and other laboratory indices, the INRS remained a significant predictor (Supplementary Table 5), confirming its stability and potential as a standalone biomarker. Patient distributions according to INRS categories are illustrated in Figure 3A–C.

### Independent risk factors for RBE

**Figure 4.**
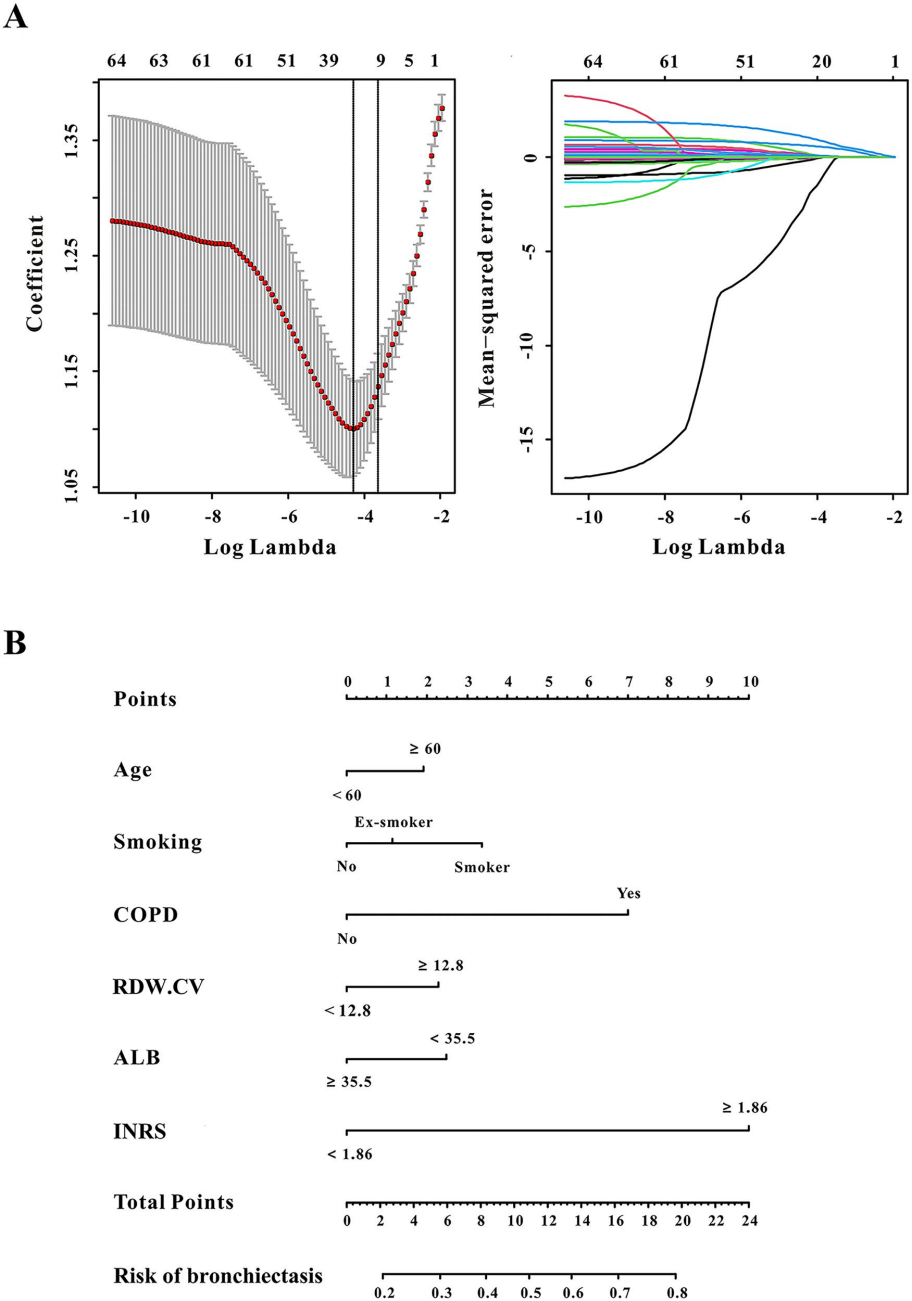
The RBE nomogram model construction. (A) The LASSO regression for variables selection; (B) Nomogram model for predicting RBE in patients after TBI.

Fourteen candidate predictors selected by LASSO regression (Figure 4A) were entered into univariate analysis. Subsequent multivariate logistic regression identified six independent risk factors for RBE (Table 3): age ≥60 years (OR = 1.19, 95% CI: 1.05–1.78; *p* = 0.030), current smoking (OR = 1.71, 95% CI: 1.14–2.56; *p* = 0.009), COPD (OR = 3.13, 95% CI: 2.30–4.26; *p* < 0.001), RDW-CV ≥12.8% (OR = 1.09, 95% CI: 1.03–1.16; *p* = 0.005), albumin <35.5 g/L (OR = 1.04, 95% CI: 1.01–1.06; *p* = 0.003) and INRS ≥1.86 (OR = 5.04, 95% CI: 3.79–6.70; *p* < 0.001).

### Development and validation of the RBE nomogram model

A clinical nomogram incorporating the six independent risk factors was constructed to predict individual RBE risk (Figure 4B). The model exhibited excellent and consistent discriminative ability, with AUCs of 0.839, 0.837 and 0.829 in the development, internal validation and external validation sets, respectively (Figure 2A–C). The DeLong test showed no significant differences in AUC between the development set and internal validation set (*p* = 0.913) or external validation set (*p* = 0.351), confirming robust discriminative performance across cohorts.

Calibration curves indicated strong agreement between predicted and observed RBE probabilities across all cohorts (Figure 2D–F). Calibration performance was comprehensively evaluated using calibration curves and calibration parameters (intercept and slope). In the training set, the calibration curve showed strong agreement between predicted and observed RBE probabilities (Figure 2D), with a calibration intercept of 0.000, and slope of 1.000 (close to the ideal value of 1). In the internal validation set (Figure 2E), the calibration intercept was 0.198, and slope was 0.832, indicating mild overestimation of low risks and underestimation of high risks but overall acceptable calibration. For the external validation set (Figure 2F), the external calibration intercept was 0.148, and slope was 1.151; although the slope deviated slightly from the ideal value (1.0), the predicted and observed risks remained moderately consistent, and no recalibration was performed as the deviation did not substantially affect clinical utility.

DCA further confirmed the clinical utility of the nomogram. Compared to the INRS alone, the nomogram provided greater net benefit across a wide range of threshold probabilities in all validation sets, supporting its superior value for clinical decision-making (Figure 2G–I).

## Discussion

Radiological bronchiectasis (RBE) is a recently codified imaging entity, defined by international consensus as bronchiectasis identified on computed tomography in the absence of typical clinical symptoms [2]. As research advances, RBE has garnered increasing attention across various respiratory diseases, including TB [16], cystic fibrosis [23], lung cancer [24] and COPD [25]. Nevertheless, studies focusing specifically on RBE following TBI remain scarce. In our cohort from Wuhan, China, the incidence of RBE after TBI was 42.33%, aligning closely with the approximate 40% reported in India [16].

Accumulating evidence links systemic inflammation and nutritional status to clinically evident bronchiectasis (CBE) [26–33]. Studies, primarily from China, report elevated inflammatory markers (e.g. TNF-α, fibrinogen, ESR, CRP, ceruloplasmin) and reduced nutritional parameters (e.g. albumin, pre-albumin, BMI, fat-free mass index) in patients with mild-to-moderate CBE compared to healthy controls [26–28]. Furthermore, higher systemic inflammation correlates with greater disease severity in CBE [26,29–32] while better nutritional status may attenuate severity, particularly in females [33]. However, due to its asymptomatic nature, the mechanisms and risk factors for RBE are poorly characterized [2,20]. A recent study investigating bronchial gene expression in RBE proposed a novel mechanistic pathway involving enhanced ciliary activity and downregulation of Wnt signaling [34]. Notably, no prior literature has explored the aetiology or risk factors for RBE specifically after TBI. Given China’s high TB burden, elucidating these factors is crucial for developing personalized post-TB management strategies.

Consistent with the inflammatory-nutritional paradigm, our study found significantly higher levels of SII, NHR, MHR, PHR, MRR, CAR, CPR and CONUT scores, alongside lower PNI and HALP scores, in the RBE group compared to the NRBE group. Multivariate analysis confirmed that lower PNI, lower HALP, higher Lg(SII) and higher CAR were the independent risk factors for RBE after TBI. These findings align with prior work on CBE [26–28,34] and suggest that inflammatory activation and nutritional depletion occur early in BE pathogenesis. We hypothesize that sustained inflammation and malnutrition may drive the progression from asymptomatic RBE to symptomatic CBE. This is supported by our earlier finding that TB patients with RBE have reduced survival [16]. To enable early risk stratification, we developed the INRS, which demonstrated moderate but consistent predictive performance across all cohorts (AUCs: 0.757–0.761). Seeking improved accuracy, we integrated the INRS with key clinical variables to construct a more robust predictive nomogram.

The final model identified six independent risk factors: age ≥60 years, current smoking, COPD, RDW-CV ≥12.8%, albumin <35.5 g/L and INRS ≥1.86. The association with advancing age is consistent with epidemiology of CBE [35] and may be explained by age-related immunosenescence [36] and the cumulative impact of comorbidities on nutritional and inflammatory homeostasis. Smoking, more prevalent in Indian patients with post-TB bronchiectasis [37] and a known risk factor for RBE in other contexts [38], likely promotes bronchial inflammation *via* nicotine-induced release of TNF-α [39]. The strong link with COPD underscores shared pathophysiological pathways, particularly neutrophilic inflammation and impaired local immunity, which perpetuates airway damage and remodeling [40]. Hypoalbuminaemia, a marker of nutritional deficit and systemic inflammation, confirmed its role as a risk factor, echoing findings in CBE [27]. While RDW-CV has been linked to poor outcomes in anaemic TB patients [35], our study is the first to report its independent association with RBE risk after TBI, highlighting its potential as a novel haematological biomarker.

Finally, the prediction nomogram model was successfully constructed to identify the high-risk population of RBE after TBI. The prediction nomogram model showed an excellent predictive value, with AUC of 0.839, 0.837 and 0.829 in the development cohort, internal validation cohort and external validation cohort, respectively. In addition, the INRS-based nomogram model also showed a strong accuracy and clinical utility in development, internal validation and external validation cohorts. To our knowledge, only one prior study has developed a predictive model for RBE progression to CBE [41]. Our work is therefore novel in being the first to develop and validate an inflammation-nutrition-based score and a comprehensive nomogram for predicting RBE incidence after TBI.

This model offers several translational advantages. First, it integrates pathogenic mechanisms (inflammation–nutrition imbalance) with readily available clinical parameters, enabling targeted interventions such as smoking cessation, nutritional support and anti-inflammatory management. Second, all included variables are routinely accessible even in primary care settings, making the tool particularly valuable for resource-limited, high-TB-burden regions. Finally, its high predictive accuracy can assist in triaging high-risk individuals for earlier referral and specialized follow-up, potentially reducing the incidence of progressive lung disease, associated healthcare costs and improving long-term quality of life. However, our study has several limitations. First, all participants were from Wuhan; the generalizability of the INRS to other populations requires further validation. Second, we could not fully account for the potential confounding effects of anti-tuberculosis therapy on inflammatory and nutritional biomarkers. Third, a key limitation stems from our retrospective cross-sectional design. We lack precise data on the time interval between the completion of anti-tuberculosis therapy and the chest CT scan used to diagnose RBE. Consequently, our study identifies predictors associated with the presence of RBE at a post-treatment assessment but cannot distinguish between prevalent RBE (existing at treatment completion) and incident RBE (developing during the follow-up period). This ambiguity affects the etiological interpretation of the identified risk factors. While our clinical protocol typically involved imaging during follow-up (6–24 months post-treatment), future prospective studies should rigorously record this interval to define and predict the true incident of RBE.

## Conclusion

In summary, this study is the first to develop an Inflammation-Nutrition Risk Score (INRS) based on PNI, HALP, Lg(SII) and CAR for predicting RBE after TBI. The INRS alone showed promising discriminatory ability, and its integration with clinical parameters in a nomogram yielded a tool with excellent predictive performance, calibration and clinical utility. This model provides a practical, evidence-based approach for risk stratification and personalized management of patients after tuberculosis infection, representing a step toward precision medicine in post-TB care.

## Supplementary Material

Supplementary materials_Supplementary Tables and Supplementary Figure.docx

## Data Availability

The data that support the findings of this study are available from the corresponding author(Fengyun Gong or Xiaorong Wang) upon reasonable request.

## Abbreviations

BE: bronchiectasis
BMI: body mass index
CBE: clinical BE
CCA: calibrate curve analysis
CPDH: chronic pulmonary heart disease
COPD: chronic obstructive pulmonary disease
DCA: decision curve analysis
DM: diabetes mellitus
EMR: electronic medical record
HBV: chronic hepatitis B
HTM: hypertension
INRS: inflammation-nutrition risk score
LASSO: Least Absolute Shrinkage and Selection Operator
NRBE: non-radiological bronchiectasis
RBE: radiological bronchiectasis
ROC: receiver operating characteristics
TBI: tuberculosis infection
ULSA and MLSA: Univariate and Multivariate Logistic regression analysis
