# Supplementary material for: A novel inflammation-nutrition risk score (INRS) and its related nomogram model to predict radiological bronchiectasis in patients after tuberculosis infection in Wuhan, China
Source: Ann Med. 2026 Feb 17;58(1):2625545. doi: 10.1080/07853890.2026.2625545 (PMC12918364; doi:10.1080/07853890.2026.2625545)
Supplement: Supplementary materials_Supplementary Tables and Supplementary Figure.docx [file IANN_A_2625545_SM7731.docx]

**Supplementary table 1** the computing method of inflammation and nutrition indexes

| Variables | formula | | | | |
| --- | --- | --- | --- | --- | --- |
| Inflammation indexes |  | | | | |
| SII | Platelet count (10^^9^/L) × neutrophil count(10^^9^/L)/lymphocyte count (10^^9^/L) | | | | |
| SIRI | Neutrophil count (10^^9^/L) × monocyte count (10^^9^/L)/lymphocyte count (10^^9^/L) | | | | |
| NLR | Neutrophil count (10^^9^/L)/lymphocyte count (10^^9^/L) | | | | |
| MLR | Monocyte count (10^^9^/L)/lymphocyte count (10^^9^/L) | | | | |
| PLR | Platelet count (10^^9^/L)/lymphocyte count (10^^9^/L) | | | | |
| dNLR | Neutrophils count (10^^9^/L)/ (leukocyte count (10^^9^/L) ‐neutrophil count (10^^9^/L)) | | | | |
| NHR | Neutrophil count (10^^9^/L)/high density lipoprotein (mmol/L) | | | | |
| MHR | Monocyte count (10^^9^/L)/high density lipoprotein (mmol/L) | | | | |
| PHR | Platelet count (10^^9^/L)/high density lipoprotein (mmol/L) | | | | |
| MRR | Monocyte count (10^^9^/L)/red blood cell count (10^^12^/L) | | | | |
| CAR | C-reaction protein(mg/L) / serum albumin (g/L) | | | | |
| CPR | C-reaction protein (mg/L) / serum pre-albumin (mg/L) | | | | |
| CLR | C-reaction protein(mg/L)/ lymphocyte count (10^9/L) | | | | |
| Nutrition indexes |  |  |  |  |  |
| HALP score | Hemoglobin (g/L) ×serum albumin (g/L) ×lymphocyte count (10^^9^/L)/platelet count (10^^9^/L) | | | | |
| PNI | Albumin (g/dL) + 5×Lymphocyte count (10^^9^/L) | | | | |
| COUNT score | Parameters | Normal | Light | Moderate | Severe |
|  | Serum albumin (g/L) | ≥35 | 30-34.9 | 25-29.9 | <25 |
|  | Score | 0 | 2 | 4 | 6 |
|  | Total lymphocyte (10^^9^/L) | ≥1.6 | 1.2-1.599 | 0.8-1.199 | <0.8 |
|  | Score | 0 | 1 | 2 | 3 |
|  | Total cholesterol (mmol/L) | ≥10 | 7.78-10 | 5.56-7.78 | <5.56 |
|  | Score | 0 | 1 | 2 | 3 |

**Abbreviation:** SII, Systemic Inflammation Index; SIRI, Systemic Inflammatory Response Index; NLR, Neutrophil count to lymphocyte count ratio; MLR, Monocyte count to lymphocyte count ratio; PLR, Platelet count to lymphocyte count ratio; dNLR, Neutrophils count to (leukocyte count ‐neutrophil count) ratio; NHR, Neutrophil count to high density lipoprotein ratio; MHR, Monocyte count to high density lipoprotein ratio; PHR, Platelet count to high density lipoprotein ratio; MRR, Monocyte count to red blood cell count ratio; CAR, C-reaction protein to serum albumin ratio; CPR, C-reaction protein to serum pre-albumin ratio; CLR, C-reaction protein to lymphocyte count; HALP score, hemoglobin-albumin-lymphocyte-platelet score; PNI, prognostic nutritional index; COUNT, controlling nutritional status score

**Supplementary table 2** Comparison of study variables between RBE group and NRBE group in the development set

| Variables | RBE group(n=574) | NRBE group(n=734) | P value |
| --- | --- | --- | --- |
| Age | 50.78(16.74) | 44.65(17.41) | <0.001 |
| Gender |  |  |  |
| Male | 417(72.6) | 523(71.3) |  |
| Female |  |  | 0.621 |
| weight (mean (SD)) | 54.01 (9.32) | 56.68 (10.08) | <0.001 |
| Smoking history |  |  |  |
| Smoker | 158(27.5) | 209(28.5) |  |
| Ex-smoker | 133(23.2) | 109(14.9) |  |
| Non-smoker | 283(49.3) | 416(56.7) | <0.001 |
| Drinking history |  |  |  |
| Drinker | 66(11.5) | 98(13.4) |  |
| Ex-drinker | 42(7.3) | 65(8.9) |  |
| Non-drinker | 466(81.2) | 571(77.8) | 0.32 |
| Underlying disease |  |  |  |
| HTN | 56(9.8) | 83(11.3) | 0.416 |
| DM | 112(19.5) | 132(18.0) | 0.527 |
| CPHD | 78(13.6) | 44(6.0) | <0.001 |
| COPD | 223(38.9) | 114(15.5) | <0.001 |
| HBV | 86(15.0) | 99(13.5) | 0.49 |
| Laboratory indexes |  |  |  |
| WBC(mean(SD) | 7.35(2.94) | 6.72(2.65) | <0.001 |
| RBC(mean(SD) | 4.01(0.63) | 4.11(0.63) | 0.005 |
| HGB(mean(SD) | 112.95(19.26) | 117.88(19.76) | <0.001 |
| HCT(mean(SD) | 35.23(5.46) | 36.39(5.65) | <0.001 |
| PLT(mean(SD) | 276.18(99.94) | 258.51(95.76) | 0.001 |
| MCV(mean(SD) | 88.03(7.89) | 88.79(6.58) | 0.059 |
| MCH(mean(SD) | 28.29(2.91) | 28.76(2.66) | 0.002 |
| MCHC(mean(SD) | 320.13(15.96) | 323.64(11.97) | <0.001 |
| LYM(mean(SD) | 1.15(0.54) | 1.21(0.58) | 0.043 |
| MON(mean(SD) | 0.55(0.26) | 0.51(0.24) | 0.003 |
| EOS(mean(SD) | 0.13(0.15) | 0.13(0.16) | 0.779 |
| BAS(mean(SD) | 0.05(0.57) | 0.03(0.02) | 0.217 |
| RDW.CV(mean(SD) | 13.95(2.54) | 13.34(1.81) | <0.001 |
| PDW(mean(SD) | 15.85(0.53) | 15.87(0.65) | 0.694 |
| MPV(mean(SD) | 8.96(1.29) | 9.19(1.21) | 0.001 |
| PCT(mean(SD) | 0.24(0.07) | 0.23(0.07) | 0.03 |
| PT(mean(SD) | 11.81(1.18) | 11.69(1.45) | 0.114 |
| INR(mean(SD) | 1.02(0.11) | 1.01(0.13) | 0.111 |
| APTT(mean(SD) | 31.79(6.44) | 31.92(6.36) | 0.703 |
| TT(mean(SD) | 19.10(3.76) | 19.49(4.31) | 0.088 |
| ESR(mean(SD) | 55.76(30.48) | 48.97(29.73) | <0.001 |
| TBIL(mean(SD) | 11.13(13.40) | 11.47(9.41) | 0.586 |
| DBIL(mean(SD) | 4.77(9.33) | 4.46(7.62) | 0.496 |
| AST(mean(SD) | 22.33(66.00) | 21.96(34.67) | 0.894 |
| ALT(mean(SD) | 29.71(54.73) | 28.11(30.30) | 0.501 |
| TB(mean(SD) | 65.23(7.49) | 65.83(7.26) | 0.141 |
| ALB(mean(SD) | 33.57(5.71) | 35.29(6.01) | <0.001 |
| GLB(mean(SD) | 31.68(6.40) | 30.55(6.06) | 0.001 |
| ALP(mean(SD) | 92.56(45.51) | 92.04(62.65) | 0.868 |
| GGT(mean(SD) | 41.60(47.51) | 43.23(65.25) | 0.614 |
| TBA(mean(SD) | 7.46(14.28) | 6.50(16.83) | 0.275 |
| eGFR(mean(SD) | 138.83(53.27) | 136.00(53.50) | 0.342 |
| BUN(mean(SD) | 4.69(4.19) | 4.54(2.66) | 0.427 |
| Scr(mean(SD) | 64.25(27.27) | 68.49(42.77) | 0.039 |
| UA(mean(SD) | 315.55(142.76) | 314.53(144.67) | 0.898 |
| CysC(mean(SD) | 0.97(0.50) | 0.97(0.63) | 0.92 |
| Glu(mean(SD) | 6.57(4.28) | 6.00(2.61) | 0.003 |
| CK(mean(SD) | 59.40(55.39) | 74.04(80.63) | <0.001 |
| CKMB(mean(SD) | 12.16(6.94) | 11.98(6.67) | 0.638 |
| LDH(mean(SD) | 196.93(82.67) | 197.99(111.11) | 0.85 |
| K^+^(mean(SD) | 4.10(0.51) | 4.10(0.52) | 0.971 |
| Na^+^(mean(SD) | 138.09(4.22) | 138.31(4.06) | 0.329 |
| Cl^-^(mean(SD) | 102.56(4.49) | 103.38(4.30) | 0.001 |
| Ca^2+^(mean(SD) | 2.17(0.16) | 2.19(0.16) | 0.028 |
| ADA(mean(SD) | 16.01(7.59) | 15.96(9.20) | 0.926 |
| aHBDH(mean(SD) | 131.58(62.42) | 132.65(64.11) | 0.761 |
| PALB(mean(SD) | 122.32(55.04) | 137.22(59.32) | <0.001 |
| TC(mean(SD) | 3.79(0.88) | 3.80(0.90) | 0.855 |
| TG(mean(SD) | 1.03(0.60) | 1.06(0.61) | 0.475 |
| LDL(mean(SD) | 2.02(0.83) | 1.90(0.84) | 0.01 |
| HDL(mean(SD) | 1.37(0.77) | 1.49(0.83) | 0.006 |
| SAA(mean(SD) | 114.09(121.62) | 103.81(95.82) | 0.088 |
| CRP(mean(SD) | 53.29(43.77) | 38.89(40.29) | <0.001 |
| **Inflammation indexes** |  |  |  |
| SII(mean(SD) | 2130.77(2477.68) | 1106.38(852.41) | <0.001 |
| SIRI(mean(SD) | 3.91(4.82) | 3.34(10.74) | 0.238 |
| NLR(mean(SD) | 6.84(7.53) | 6.25(15.64) | 0.408 |
| MLR(mean(SD) | 0.60(0.47) | 0.54(0.64) | 0.059 |
| PLR(mean(SD) | 312.14(251.94) | 288.37(381.96) | 0.198 |
| dNLR(mean(SD) | 3.42(4.73) | 3.14(3.23) | 0.196 |
| NHR(mean(SD) | 5.93(6.10) | 4.89(4.71) | 0.001 |
| MHR(mean(SD) | 0.57(0.45) | 0.50(0.36) | 0.001 |
| PHR(mean(SD) | 280.28(205.21) | 243.03(166.46) | <0.001 |
| MRR(mean(SD) | 0.14(0.07) | 0.13(0.06) | <0.001 |
| CAR(mean(SD) | 1.85(1.55) | 1.10(1.10) | <0.001 |
| CPR(mean(SD) | 0.83(1.91) | 0.60(1.99) | 0.032 |
| CLR(mean(SD) | 73.32(116.50) | 60.61(182.77) | 0.147 |
| Nutrition indexes |  |  |  |
| PNI(mean(SD) | 34.23(10.91) | 44.06(7.60) | <0.001 |
| HALP(mean(SD) | 16.12(10.80) | 28.32(66.00) | <0.001 |
| COUNT(mean(SD) | 6.46(2.56) | 5.94(2.53) | <0.001 |

**Abbreviation:** CPHD, chronic pulmonary heart disease; COPD, chronic obstructive pulmonary disease; PNI, prognostic nutritional index; HALP score, hemoglobin-albumin-lymphocyte-platelet score; COUNT score, controlling nutritional status score; SII, Systemic Inflammation Index; NHR, Neutrophil count to high density lipoprotein ratio; MHR, Monocyte count to high density lipoprotein ratio; PHR, Platelet count to high density lipoprotein ratio; MRR, Monocyte count to red blood cell count ratio; CAR, C-reaction protein to serum albumin ratio; CPR, C-reaction protein to serum pre-albumin ratio; CLR, C-reaction protein to lymphocyte count; WBC, white blood cell; RBC, red blood cell; HGB, hemoglobin; HCT, Hematocrit; PLT, platelet; MCH, mean Corpuscular hemoglobin; MCHC, mean corpuscular hemoglobin concentration; LYM, lymphocyte; MON, Monocyte; RDW-CV, red blood cell distribution width-coefficient of variation; ESR, erythrocyte sedimentation rate; ALB, albumin; GLB, globulin; Scr, serum creatinine; GLU, glucose; CK, creatine kinase; PALB, prealbumin; LDL, low density lipoprotein; HDL, high density lipoprotein; CRP, C-reactive protein.

**Supplementary table 3 VIF value in INRS**

| Variables | VIF | Tolerance |
| --- | --- | --- |
| Lg (SII) | 1.435 | 0.697 |
| CAR | 1.311 | 0.763 |
| PNI | 1.169 | 0.855 |
| HALP | 1.165 | 0.859 |

**Abbreviation:** SII, Systemic Inflammation Index; CAR, C-reaction protein to serum albumin ratio; HALP score, hemoglobin-albumin-lymphocyte-platelet score; PNI, prognostic nutritional index

**Supplementary table 4 VIF value in RBE nomogram**

| Variables | VIF | Tolerance |
| --- | --- | --- |
| Age | 1.349 | 0.741 |
| Smoking | 1.139 | 0.878 |
| COPD | 1.260 | 0.794 |
| RDW.CV | 1.057 | 0.946 |
| ALB | 1.282 | 0.780 |
| INRS | 1.135 | 0.881 |

**Abbreviation:** COPD, chronic obstructive pulmonary disease; RDW-CV, red blood cell distribution width-coefficient of variation; ALB, albumin.

**Supplementary table 5** Univariate and multivariate logistic regression analysis of INRS for RBE

| Methods | OR (95%CI) | P value |
| --- | --- | --- |
| In development set |  |  |
| Unadjusted | 4.74 (3.75-6.00) | <0.001 |
| Adjusted for model I | 4.53 (3.49-5.88) | <0.001 |
| Adjusted for model II | 4.49 (3.35-5.92) | <0.001 |
| Adjusted for model III | 6.12 (4.47-8.37) | <0.001 |
| In internal validation set |  |  |
| Unadjusted | 3.62 (2.51-5.22) | <0.001 |
| Adjusted for model I | 3.17 (2.16-4.64) | <0.001 |
| Adjusted for model II | 2.83 (1.91-4.21) | <0.001 |
| Adjusted for model III | 4.27 (2.59-7.04) | <0.001 |
| In external validation set |  |  |
| Unadjusted | 9.75 (5.61-16.92) | <0.001 |
| Adjusted for model I | 9.95 (5.60-17.68) | <0.001 |
| Adjusted for model II | 3.772 (1.93-7.39) | <0.001 |
| Adjusted for model III | 4.528 (2.25-9.12) | <0.001 |

Model I adjusted for age, gender, weight. Model II adjusted for model I plus smoking, drinking, and comorbidities. Model III adjusted for Model II plus laboratory results, OR, odds ratio, 95%CI, 95% confidence interval.

**
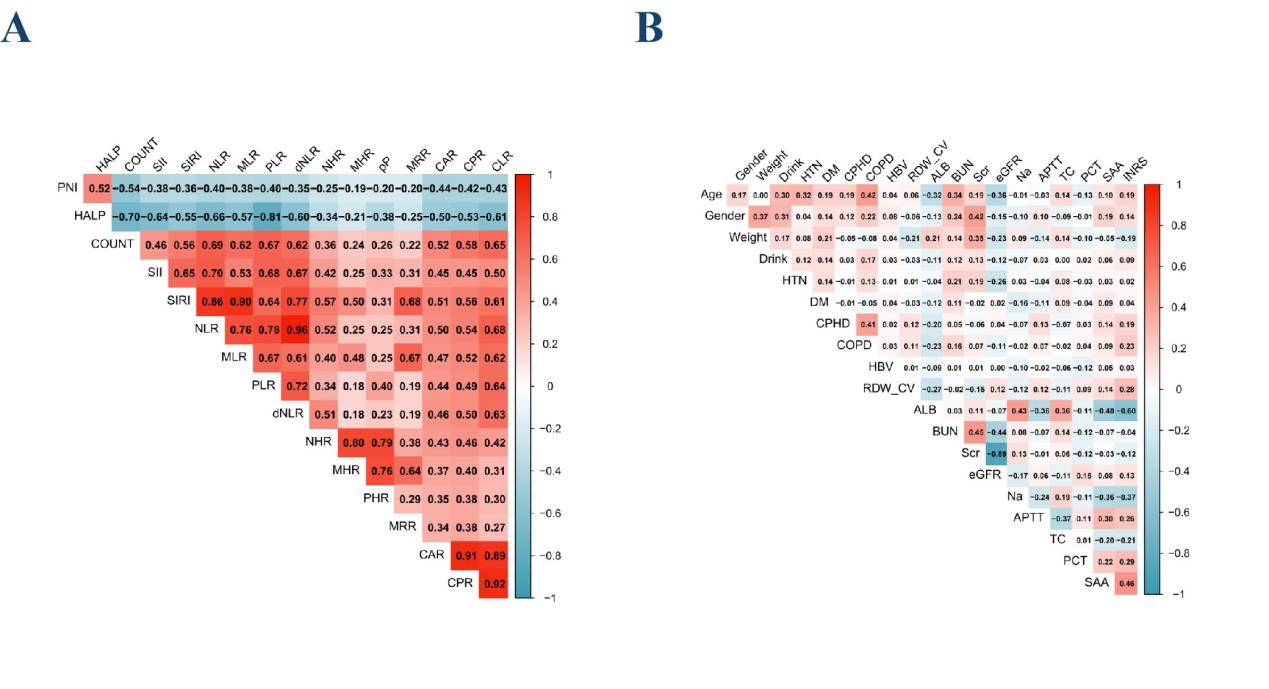
**

**Note: A-INRS; B-Nomogram**

**Supplementary Figure 1 Pearson correlation matrix of all candidate variables**
